# Specific Recognition of a Stem-Loop RNA Structure by the Alphavirus Capsid Protein

**DOI:** 10.3390/v13081517

**Published:** 2021-07-31

**Authors:** Rebecca S. Brown, Lisa Kim, Margaret Kielian

**Affiliations:** Department of Cell Biology, Albert Einstein College of Medicine, New York, NY 10461, USA; rebeccashbrown@gmail.com (R.S.B.); hyunjung.kim@einsteinmed.org (L.K.)

**Keywords:** alphavirus, nucleocapsid, capsid protein, RNA packaging, RNA binding

## Abstract

Alphaviruses are small enveloped viruses with positive-sense RNA genomes. During infection, the alphavirus capsid protein (Cp) selectively packages and assembles with the viral genomic RNA to form the nucleocapsid core, a process critical to the production of infectious virus. Prior studies of the alphavirus Semliki Forest virus (SFV) showed that packaging and assembly are promoted by Cp binding to multiple high affinity sites on the genomic RNA. Here, we developed an in vitro Cp binding assay based on fluorescently labeled RNA oligos. We used this assay to explore the RNA sequence and structure requirements for Cp binding to site #1, the top binding site identified on the genomic RNA during all stages of virus assembly. Our results identify a stem-loop structure that promotes specific binding of the SFV Cp to site #1 RNA. This structure is also recognized by the Cps of the related alphaviruses chikungunya virus and Ross River virus.

## 1. Introduction

The *Alphavirus* genus includes a number of medically important species such as chikungunya virus (CHIKV), Ross River virus (RRV), Mayaro virus, and the encephalitic alphaviruses (reviewed in [1,2,3,4,5,6]). Alphaviruses are transmitted to humans by mosquito vectors, and sporadic outbreaks and epidemics of these viruses have caused millions of human infections across the world. There are currently no approved vaccines or antiviral therapies to combat alphavirus infection, although promising candidates are under development (e.g., [7,8]).

Alphaviruses are small enveloped positive-sense RNA viruses that assemble into highly organized particles with icosahedral symmetry (reviewed in [1]). Virus assembly and budding are complex processes that involve critical interactions between several viral components [9]. To produce infectious virus particles, the capsid protein (Cp) must package the ~11.5 kb genomic RNA (gRNA) and assemble with it to form the nucleocapsid (NC). Cp binds RNA through its primarily unstructured poly-basic N-terminal domain, while its C-terminal structured domain binds the cytoplasmic domain of the E2 envelope protein [10]. Cp’s oligomerization and assembly into NC is dependent on charge neutralization contributed by the RNA [10,11] and a motif in the Cp linker region that is required for cytoplasmic NC assembly [12,13]. The infectious viral NC consists of 240 copies of the Cp protein organized around a single copy of the gRNA. 

The alphavirus Cp selectively packages the gRNA to produce virus particles with a high specific infectivity. During infection the viral RNA replication complex produces both the gRNA and a subgenomic RNA (sgRNA) encoding the viral structural proteins. However, in spite of the presence of a molar excess of sgRNA and cellular RNAs in alphavirus-infected cells, for most alphaviruses packaging strongly favors the viral genomic RNA ([14,15,16,17,18], but see also [19]). Studies with the alphavirus Semliki Forest virus (SFV) show that the mechanism of selective gRNA packaging depends on Cp binding to multiple high affinity sites that are unique to the gRNA [20]. The multiple, high affinity Cp binding sites ensure specific recognition and faithful packaging of the viral genome into new particles. This multi-site packaging mechanism may guard against deleterious mutations generated by error-prone RNA replication, and/or facilitate the co-assembly of Cp and gRNA into NC [21,22]. 

We previously demonstrated that the SFV Cp specifically recognizes its top gRNA binding site, termed site #1, across all stages of virus assembly [20]. We reconstituted this specific binding in vitro using purified Cp and ^32^P-labeled RNA, and found that site #1 RNA specifically promotes the in vitro assembly of core-like particles [20]. The specific binding to site #1 RNA suggests that this site encompasses a specific sequence and/or structural motif preferentially recognized by Cp. Both site #1 and site #2, the second best gRNA binding site in the cell, contain an identical 7 nucleotide sequence not present elsewhere in the gRNA, and are predicted to adopt a stem-loop structure [20]. Here, we used in vitro binding studies to dissect the requirements for the recognition of site #1 RNA by the alphavirus Cp. Our results show that the SFV Cp recognizes a specific stem-loop structure, and that this recognition mechanism is conserved for other alphavirus Cps. 

## 2. Materials and Methods

### 2.1. Cp Expression and Purification

SFV, RRV and CHIKV Cp expression vectors were constructed as previously described [20] in pET29a with an N-terminal double Strep Tag (WSHPQFEK) followed by a glycine + serine linker and a TEV protease cleavage site. The SFV and RRV Cp were expressed in Rosetta2 bacteria as previously described [20], and purified by affinity chromatography using Strep-Tactin sepharose (IBA Lifesciences, Goettingen, Germany). The CHIKV Cp was expressed by inducing Rosetta2 cells at an OD_600_~0.5 for 4 h at 37 °C [23], and purified as for the RRV and SFV Cps except using twice the starting material. 1 µg of each protein was subjected to SDS-PAGE followed by Coomassie staining to assess protein integrity and purity. 

### 2.2. Alexa488-RNAs

HPLC-purified 5′ Alexa488-labeled RNA oligos were purchased from Integrated DNA Technologies (Coralville, IA, USA). RNAs were resuspended in 50 mM Tris pH 7.0, aliquoted, and snap frozen. Then, 50 ng of each RNA was subjected to 15% denaturing Urea-PAGE followed by staining with SYBR Gold (Invitrogen, Waltham, MA, USA) to confirm RNA integrity. Site #1 RNA was previously mapped to nt 5988–6016 in the SFV gRNA [20]. 

### 2.3. RNA Binding Assay

Black, non-binding 96-well plates (Corning, Corning, NY, USA) were washed twice with Buffer W (100 mM Tris pH 8.0, 150 mM NaCl, 1 mM EDTA). MagStrep “type3” XT beads (IBA Lifesciences, Goettingen, Germany) were equilibrated in Buffer W and added to each well to capture Strep-tagged Cp. Cp was 2-fold serially diluted (4 µM to 3.9 nM) in Buffer W for n + 1 samples, added to its corresponding wells, and incubated with MagStrep “type3” XT beads for 4–6 h at 4 °C with rocking. A no Cp control was included for each RNA sample to assess background for each experiment. Plates were magnetized and washed three times with Buffer W before adding RNA Binding Buffer (25 mM Tris pH 7.0, 150 mM KCl, 3 mM MgCl_2_, 0.01% Tween-20, 1 µg/µL BSA, 1 mM DTT) to each well. Where indicated, samples contained 200 ng/µL of poly(I:C) (InvivoGen, San Diego, CA, USA) in a 50 µL volume. Samples were preincubated at 37 °C for 15 min with shaking. Alexa488-RNAs were diluted in RNA Binding Buffer, added to each well at a 10 nM final concentration, and incubated in the dark for 30 min at 37 °C with shaking. Plates were magnetized and washed three times with Wash Buffer (25 mM Tris pH 7.0, 150 mM KCl, 0.5 mM EDTA, 0.01% Tween-20). Elution Buffer (10 mM Tris pH 7.0, 1 mM EDTA, 1% SDS) was then added to each well and incubated in the dark at 70 °C for 10 min with shaking. Plates were magnetized, the supernatant was transferred to a new 96-well plate for measurement, and bubbles were removed. Fluorescence corresponding to bound Alexa488-RNA was measured on a Victor X5 plate reader (PerkinElmer, Waltham, MA, USA) using the “Fluorescein 485–535 1s” default protocol. Technical duplicates were performed for all samples. 

### 2.4. RNA Fraction Bound Calculations

Control RNAs: for each experiment, a known concentration of each Alexa488-labeled RNA was diluted in Elution Buffer in triplicate to determine the fluorescence corresponding to maximal RNA binding. Background fluorescence from the buffer alone control was negligible but was still subtracted from the individual fluorescence of the control RNAs. Background fluorescence from Cp was also negligible. The triplicate measurements of each control RNA were then averaged to calculate maximum fluorescence of the respective RNA. 

Experimental RNAs: “Fraction Bound %” corresponding to the amount of Alexa488-RNA bound at each Cp concentration was calculated by first subtracting the average background fluorescence of the “no Cp control” from each experimental sample. Experimental sample fluorescence was then normalized to the maximum fluorescence of each respective RNA (see above). This was then multiplied by one hundred to achieve “Fraction bound %”. Technical duplicates were averaged before averaging the *n* = 3 independent experiments and calculating the standard deviation. 

## 3. Results

### 3.1. Development of an In Vitro Cp-RNA Binding Assay 

**Figure 1:**
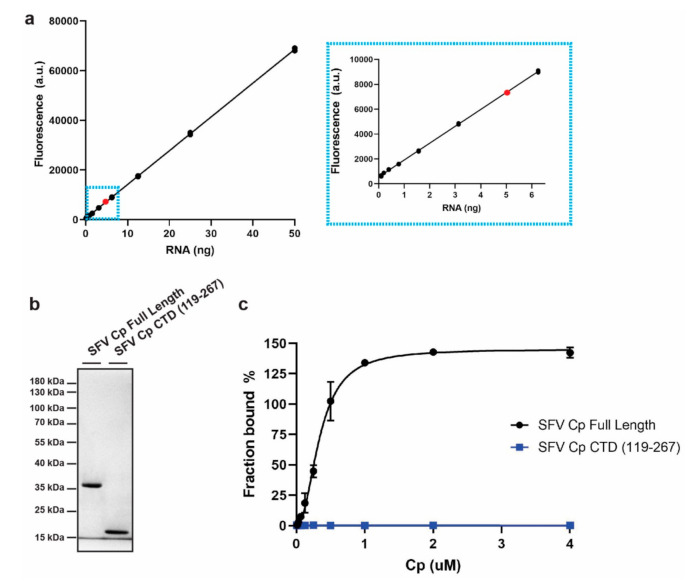
An in vitro binding assay to measure alphavirus Cp binding to fluorescently labeled RNA. (**a**) Fluorescence signal across serial dilutions of Alexa488-labeled RNA. Inset: zoom in on lower RNA quantities. Red dot represents the RNA quantity used in subsequent binding experiments. Individual data points from *n* = 2 experiments are shown. Line represents their mean. (**b**) 1 µg of purified SFV full length Cp or the Cp C-terminal domain (CTD) was subjected to SDS-PAGE and visualized by Coomassie staining. Image is representative of *n* = 5 SFV full length Cp and *n* = 1 SFV Cp CTD purifications. (**c**) Binding of increasing amounts of full length SFV Cp or SFV Cp CTD to 10 nM Alexa488-RNA. Data points represent the average from *n* = 2 independent experiments and bars represent the range. Data points are fit with nonlinear regression specific binding with Hill slope in Prism.

To identify the sequence and/or structural features that Cp recognizes within site #1 RNA, we established an in vitro Cp binding assay using fluorescently labeled RNAs (Figure 1). The assay was based on HPLC-purified, Alexa488 5′-labeled RNA. Fluorescence of the RNA was linear across a wide range of RNA amounts (Figure 1a). We recombinantly expressed and purified N-terminally Strep-tagged SFV Cps, producing either full length Cp or the Cp C-terminal domain (CTD) lacking the RNA-binding region (Figure 1b). The purified Cps were immobilized on Strep-Tactin beads at concentrations from 3.9 nM to 4 µM. Immobilization on the Strep-Tactin beads ensures that Cp remains monomeric and prevents core-like particle assembly in the presence of the added RNAs. The beads were incubated with 10 nM Alexa488-RNA and washed to remove unbound RNA. The bound RNA was then eluted, and the fluorescence was quantitated on a plate reader. Fluorescence of the input amount of Alexa488-RNA was measured in parallel to determine the fluorescence corresponding to maximal RNA binding. Fluorescence from each eluted sample was then normalized to the maximal RNA binding to determine the fraction of input RNA bound at each protein concentration (see Methods for more details). The results show that full length SFV Cp robustly bound Alexa488-RNA and that RNA binding was saturated as Cp concentrations increased (Figure 1c). In contrast, the Cp CTD did not bind RNA even at the highest Cp concentration tested (Figure 1c). This result is in agreement with the known role of the Cp N-terminal domain in binding RNA [10]. Thus, we have established a sensitive fluorescence-based assay for Cp-RNA binding that enables higher throughput and more facile laboratory handling compared to the previous ^32^P-based binding assay [20]. 

### 3.2. Cp Binding to Site #1 and Site #1 Mutant RNAs 

**Figure 2:**
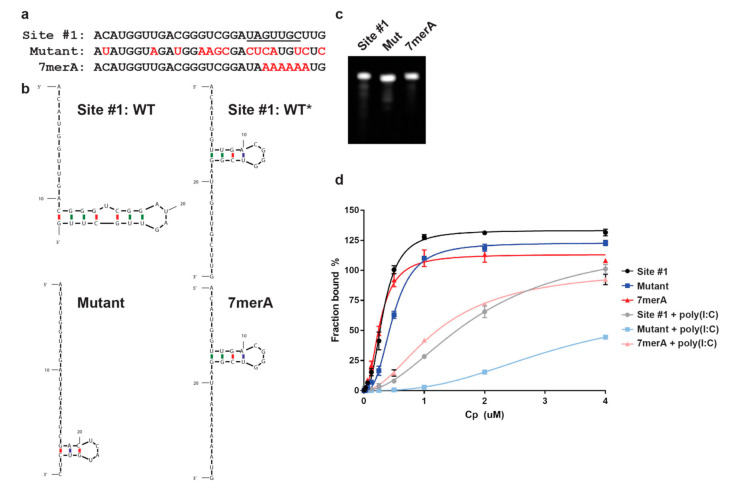
SFV Cp specifically binds site #1 and 7merA RNAs. (**a**) Sequence alignment of site #1, Mutant, and 7merA RNAs. Red nucleotides indicate mutated residues. Underlined residues are identical in Cp’s second best gRNA binding site [20]. (**b**) mFold secondary structure predictions of site #1 (WT, WT*), Mutant, and 7merA RNAs. The predicted stem loop structures in WT* and 7merA are the same. WT, WT*, Mutant, and 7merA ∆G = −0.70, −0.70, −2.50, and −0.70 kcal/mol, respectively. mFold predictions were performed as described previously [20]. (**c**) 50 ng of site #1, Mutant, and 7merA RNAs analyzed by Urea-PAGE and SYBR Gold. (**d**) SFV Cp binding to Alexa488-labeled site #1, Mutant, and 7merA RNAs, −/+ poly(I:C). Data points represent the average from *n* = 3 independent experiments. Error bars represent the standard deviation. Data points are fit with nonlinear regression specific binding with Hill slope in Prism.

We then compared Cp binding to the Alexa488-site #1 RNA vs. to Alexa488-site #1 mutant RNA (here indicated as Mutant) (Figure 2a). The Mutant is based on our previously described studies [20], and includes all possible nucleotide changes within site #1 that could be made without altering the amino acid sequence. Structure predictions indicate that these mutations would disrupt the predicted stem loop structure (termed WT) in the site #1 RNA (Figure 2b). As we previously observed using the ^32^P-labeled RNA assay [20], Cp bound both site #1 and Mutant RNAs (Figure 2d). However, when non-specific electrostatic interactions were masked by preincubating Cp with the non-specific electrostatic competitor poly(I:C), Cp showed robust binding to site #1 RNA and not to the Mutant RNA (Figure 2d). In agreement with our previous binding experiments, this result highlights Cp’s ability to not only bind nucleic acids through simple electrostatic interactions, but also through a specific recognition mechanism, and further validates our fluorescence-based binding assay. We then tested whether the conserved 7 nucleotide sequence within site #1 was required for Cp’s recognition of site #1 RNA. We mutated the 7 nucleotides to adenines (A, termed 7merA) (Figure 2a), because our previous PAR-CLIP analyses demonstrated that the Cp binding sites are relatively A-poor [20]. The 7merA RNA was predicted to adopt a short stem-loop structure distinct from the WT structure (Figure 2b). However, contrary to our hypothesis, Cp also showed robust and specific binding to the 7merA RNA in the presence of poly(I:C) (Figure 2d), demonstrating that the 7 nucleotides are not important for site #1 RNA recognition by Cp. In fact, in the presence of poly(I:C) Cp showed similar binding to 7merA RNA and site #1 RNA (Figure 2d). Further analysis by structural prediction suggested that site #1 RNA can adopt an alternative stem-loop structure that we termed WT* (Figure 2b, WT vs. WT*). WT* contains the identical short stem-loop structure/sequence predicted for the 7merA RNA (Figure 2b, WT* vs. 7merA). The WT vs. WT* site #1 structures are predicted to be equally energetically favorable and likely exist in equilibrium. In contrast, the 7merA RNA is predicted to only adopt the single structure shared with WT* (Figure 2b). Thus, the robust Cp binding to 7merA RNA appears to reflect a preference for Cp binding to the short stem-loop structure in WT* that appears to be stabilized in the 7merA RNA.

### 3.3. Role of the Predicted WT* Stem-Loop 

**Figure 3:**
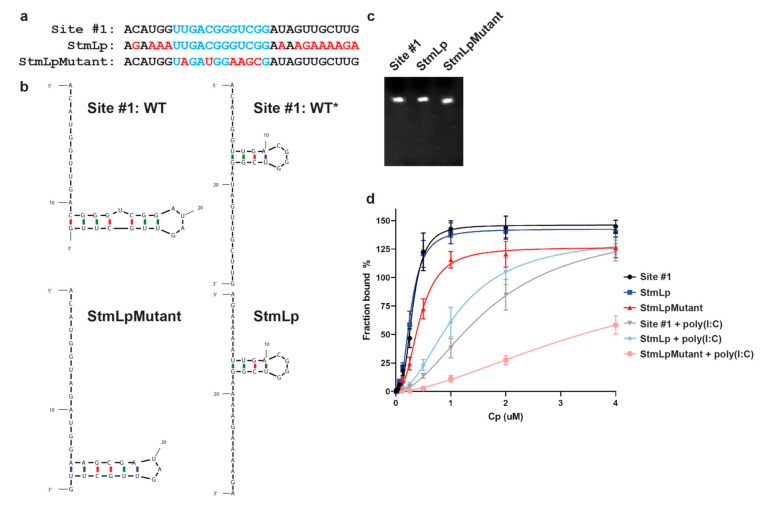
SFV Cp specifically binds a stem loop found within site #1 RNA. (**a**) Sequence alignment of site #1, StmLp, and StmLpMutant RNAs. Red nucleotides indicate mutated residues. Blue nucleotides indicate residues that make up the predicted stem loop in the WT* structure. (**b**) mFold secondary structure predictions of site #1 (WT, WT*), StmLp, and StmLpMutant RNAs. The predicted stem loop structures in WT* and StmLp are the same, and are disrupted in the StmLpMutant. WT, WT*, StmLp, and StmLpMutant ∆G = −0.70, −0.70, −0.80, and −3.50 kcal/mol, respectively. (**c**) 50 ng of site #1, StmLp, and StmLpMutant RNAs analyzed by Urea-PAGE and SYBR Gold. (**d**) SFV Cp binding to Alexa488-labeled site #1, StmLp, and StmLpMutant RNAs, −/+ poly(I:C). Data points represent the average from *n* = 3 independent experiments. Error bars represent the standard deviation. Data points are fit with nonlinear regression specific binding with Hill slope in Prism.

To test whether Cp preferentially binds the predicted WT* stem-loop, we designed two additional RNAs termed StmLp RNA and StmLpMutant RNA. The StmLp RNA maintains identity with the original site #1 sequence within the WT* stem loop, but is mutated at every other position (Figure 3a,b). The StmLpMutant RNA is mutated within the WT* stem loop, but maintains identity with the site #1 sequence at every other position (Figure 3a,b). Based on the structural predictions for these mutants (Figure 3b), if Cp preferentially recognizes the WT* stem loop, then it should specifically bind the StmLp RNA and not the StmLpMutant RNA. We tested Cp binding to Alexa488-labeled site #1, StmLp, and StmLpMutant RNAs (Figure 3c). In the presence of poly(I:C), Cp specifically bound the site #1 and StmLp RNAs, but showed weak binding to StmLpMutant RNA (Figure 3d), supporting our hypothesis that the WT* stem-loop defines Cp’s recognition of site #1. Similar to the 7merA RNA, Cp also bound the StmLp RNA with higher affinity than its binding to site #1 RNA. This is in agreement with our hypothesis that Cp specifically recognizes the WT* stem-loop adopted by the site #1, 7merA, and StmLp RNAs, and that the WT* structure may be in equilibrium with the WT structure.

### 3.4. Binding of Other Alphavirus Cps to Site #1 RNA 

**Figure 4:**
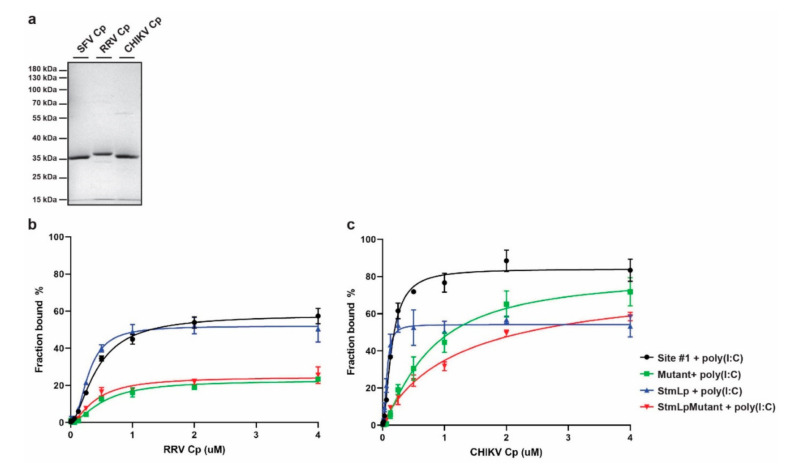
Specific recognition of site #1’s stem loop is conserved across other SFV complex alphavirus Cps. (**a**) 1 µg of SFV, RRV, and CHIKV purified Cps were subjected to SDS-PAGE and visualized by Coomassie staining. Image is representative of *n* = 5 SFV, *n* = 1 RRV, and *n* = 3 CHIKV Cp purifications. (**b**) RRV Cp binding to Alexa488-labeled site #1, Mutant, StmLp, and StmLpMutant RNAs in the presence of polyIC. Data points represent the average from *n* = 3 independent experiments. Error bars represent the standard deviation. Data points are fit with nonlinear regression specific binding with Hill slope in Prism. (**c**) As in (**b**), but for CHIKV Cp.

We next tested whether Cp’s recognition of site #1 RNA is conserved among other Cps from the SFV complex. We recombinantly expressed and purified N-terminally Strep-tagged RRV and CHIKV Cps (Figure 4a). We tested their binding to Alexa488-site #1, Mutant, StmLp, and StmLpMutant RNAs in the presence of the poly(I:C) competitor. The RRV Cp specifically bound the site #1 and StmLp RNAs, but not the Mutant and StmLpMutant RNAs (Figure 4b). This mimics SFV Cp’s RNA binding pattern and demonstrates a conserved RNA recognition mechanism between the SFV and RRV Cps. Similar to the SFV Cp, the RRV Cp bound StmLp RNA with higher affinity than the site #1 RNA. Interestingly, RRV Cp’s maximum binding to all the tested RNAs was considerably reduced compared to SFV Cp. This suggests that while the underlying mechanism governing RNA binding specificity is conserved between RRV and SFV Cps, the proteins may engage best with their cognate genomic RNA sequences. 

We then tested CHIKV Cp binding to the site #1, Mutant, StmLp, and StmLpMutant RNAs. In the presence of poly(I:C), CHIKV Cp specifically bound the site #1 and StmLp RNAs, but not the Mutant and StmLpMutant RNAs (Figure 4c). This is in agreement with the SFV and RRV Cp RNA binding patterns and further demonstrates a conserved RNA recognition mechanism within the SFV complex. As observed for the SFV and RRV Cps, CHIKV Cp bound StmLp RNA with higher affinity than site #1 RNA. However, CHIKV Cp’s maximum binding to StmLp RNA was lower than to site #1 or Mutant RNA. As the CHIKV Cp concentration increased, Mutant and StmLpMutant RNAs increasingly bound more protein, but the StmLp RNA did not. Overall, these results demonstrate that Cp’s recognition of site #1’s WT* structure is conserved across the SFV complex of alphaviruses.

## 4. Discussion

To further our understanding of the molecular mechanism of RNA recognition by the alphavirus Cp, we established an in vitro binding assay using fluorescently labeled RNAs and purified SFV Cp. N-terminally Strep-tagged full length SFV Cp was immobilized on beads so that monomeric Cp binding to RNA could be measured in the absence of confounding effects on core-like particle assembly. Using a panel of rationally designed mutated RNAs, we demonstrated that SFV Cp specifically recognized a stem-loop structure within its top genomic RNA binding site, site #1 RNA. This recognition mechanism was conserved across the alphavirus SFV complex, as RRV and CHIKV Cps also specifically recognized the stem-loop structure. 

Site #1 is predicted to adopt two structures, WT and WT*, and our data indicated that the WT* stem loop structure is specifically recognized by Cp. Both the WT and WT* secondary structures are predicted to form with nearly equivalent free energies, suggesting that they exist in equilibrium. Regulating the equilibrium and formation of the WT* structure could represent a way of regulating Cp binding to site #1 when competing RNAs and proteins are present, as is the case in the cytoplasm of an infected cell. Competing structures could also modulate Cp binding to its other top binding sites on the gRNA, and together these structural features could help to regulate when genome packaging and NC assembly occur. Additional studies will be needed to test this theory. 

Site #1’s sequence is hyper-conserved between SFV and both RRV (86%) and CHIKV (93%) compared to the overall nucleotide sequence conservation between the respective genomes (SFV and RRV: 70%; SFV and CHIKV: 67%). A comparable hyper-conservation was also found for a majority of SFV Cp’s top binding site sequences in the gRNA [20]. Thus, a conserved mechanism may govern Cp recognition of its genomic RNA binding sites across alphavirus species. While their maximal levels of binding differed, the SFV, RRV, and CHIKV Cps all displayed specific binding to the WT* stem-loop structure. Structural studies are needed to obtain a deeper molecular understanding of how this conserved recognition occurs. 

Our in vitro binding data strongly support Cp’s specific recognition of the WT* stem-loop structure within the site #1 RNA. However, the true structure of site #1 RNA within the context of the SFV genomic RNA in the complex cellular environment remains to be defined. Recent RNA structure analyses by SHAPE-MaP have been performed on alphavirus RNAs including that of CHIKV [24,25]. The conserved site #1 sequence in the CHIKV RNA lies in a region identified as structured in the SHAPE-MaP analysis. These studies used genomic RNAs purified from virus and refolded in vitro. While a significant and important advance for the field, it is as yet unclear how these SHAPE-MaP structures correlate with the genome’s structure in cells, and there are no published In-Cell SHAPE-MaP data to date. Thus, additional research is needed to further understand how the alphavirus genome’s structure may impact Cp binding and genome packaging in the context of a cellular infection.
